# Storage temperature as a determinant of probiotic long-term stability in commercial dietary supplements

**DOI:** 10.3389/fnut.2026.1933900

**Published:** 2026-09-17

**Authors:** Tereza Brousilova, Eva Vlkova, Vera Neuzil-Bunesova, Sarka Musilova, Nikol Kadlec-Modrackova

**Affiliations:** Department of Microbiology, Nutrition and Dietetics, Faculty of Agrobiology, Food and Natural Resources, Czech University of Life Sciences Prague, Prague, Czechia

**Keywords:** colony-forming units, microbial viability, probiotic bacteria, shelf-life stability, storage environment

## Abstract

**Background:**

Fueled by growing consumer demand for health-promoting interventions, probiotics have become widely used dietary supplements. Product quality assessment, however, still relies predominantly on label claims, highlighting the need to verify the presence and viability of declared microorganisms. Furthermore, the lack of harmonized methodologies and defined storage conditions complicates stability assurance and contributes to frequent discrepancies between declared and experimentally determined viable counts at the point of consumption. To address these gaps, we conducted a 12-month longitudinal evaluation of commercial multi-strain probiotic supplements stored under ambient, refrigeration, and freezing conditions, using culture-based enumeration of viable microbial counts.

**Results:**

Storage temperature was the primary determinant of viability. Under ambient storage conditions, a trend toward the greatest losses was observed, whereas lower storage temperatures appeared to better preserve bacterial counts over time. Taxon-specific patterns did not allow an unequivocal ranking of refrigeration versus freezing. Survival among bifidobacteria was strongly taxon-dependent, with *Bifidobacterium animalis* subsp. *lactis* exhibiting markedly higher technological robustness than other bifidobacterial taxa. Compliance with label-declared counts declined toward the end of minimum durability, and some products failed to meet declared values irrespective of storage regime, indicating formulation-intrinsic instability. Moreover, inconsistencies in taxonomic nomenclature and limited strain-level labeling were observed across all products.

**Conclusion:**

Overall, our findings show that cold storage conditions, specifically refrigeration and freezing, most effectively preserve probiotic viability over time. These results underscore the importance of aligning formulation strategies, including strain selection, excipient composition, and encapsulation, with intended storage conditions to ensure sustained viability and consistent product quality throughout shelf life.

## 1 Introduction

Modern society is currently facing an unprecedented increase in non-communicable civilization diseases (1), largely driven by environmental factors, sedentary lifestyle, and dietary changes, which has intensified focus on preventive health strategies. Gut homeostasis is then recognized as a significant factor in overall health, and probiotic supplementation is one potential strategy to support it (2). Probiotics are defined as live mono- or mixed cultures of microorganisms that, when administered in adequate amounts, confer health benefits on the host by modulating the composition and activity of the gut microbiota (3). Commonly used microorganisms include lactic acid bacteria, bifidobacteria, and *Saccharomyces cerevisiae* var. *boulardii* (4). Their effects involve pathogen exclusion, antimicrobial production, reinforcement of the intestinal barrier, and immunomodulation (5–7). These benefits are dependent on the delivery of sufficient numbers of viable cells, requiring stability throughout storage and shelf life (3). Probiotic products are predominantly formulated as capsules, tablets, or sachets containing lyophilized powder (8), usually supplemented with protective carriers, such as disaccharides, maltodextrin, or inulin, to enhance stability and moisture protection (9, 10). Although primarily marketed as dietary supplements, certain well-characterized probiotic strains with proven therapeutic effects have even reached the status of registered medicinal products (3).

This growing reliance on probiotics is reflected in sustained global market expansion. According to the International Probiotics Association (IPA Europe, 2022), the European probiotic supplements market reached EUR 1,464 million in 2021, accounting for nearly 25% of global value, with projected growth of 3% through 2026.

Despite this great interest, it is striking that EU regulation regarding probiotics remains fragmented. While existing legislation requires declared amounts of physiologically active substances to be maintained until the end of shelf life, no binding storage conditions are specified, placing full responsibility on manufacturers. This is compounded by Regulation (EC) No. 1924/2006, under which the term “probiotic” is considered an unauthorized health claim in most Member States. The lack of a harmonized legal category and standardized analytical oversight has resulted in frequent discrepancies between declared and actual CFU counts at consumption (11–14).

Current probiotic quality assessment relies primarily on two approaches. Culture-based methods remain essential for determining viable microorganisms and enabling strain-level characterization, but are limited by low taxonomic resolution, long analysis times, and inability to detect viable but non-culturable (VBNC) cells. Furthermore, standardized cultivation protocols are often designed for other matrices, reducing their applicability to probiotic supplements, while available media may not support the full range of commercially relevant species (15). In contrast, culture-independent molecular methods offer higher sensitivity and rapid analysis. Techniques such as multiplex PCR, Multiplex oligonucleotide ligation-PCR, or Multi-Analyte Profiling (xMAP) technology allow for the simultaneous identification and quantification of multiple species, even within multi-strain formulations (16–18). However, these methods require substantial capital investment and, most importantly, do not inherently distinguish between viable and dead cells, which is necessary for evaluating the physiological state of probiotics at the time of consumption.

Despite limitations, the plate count method remains the “gold standard” for the probiotic industry (15). International standards rely on CFU-based enumeration for probiotics and contaminants (19, 20). Its continued use reflects its direct alignment with clinical trials, which define probiotic dosage almost exclusively in CFU units (21). Established literature suggests that a minimum of 10^9^ live cells per daily portion is generally required to produce a significant health effect (3, 4). Nevertheless, a different daily dose may be accepted if substantiated by specific studies demonstrating that a particular strain remains effective in smaller amounts (22).

Consequently, as health benefits are strictly dependent on the survival of viable cells reaching the colon, a systematic evaluation of product stability is essential. Therefore, the objective of this study was to verify the taxa composition and declared CFU counts of commercial probiotic supplements, and to evaluate the longitudinal impact of varying storage conditions (ambient, refrigeration, and freezing) on microbial survival. Ultimately, this work aims to identify the optimal storage parameters required to maximize bacterial viability and safeguard product quality from production to the end-user.

## 2 Materials and methods

### 2.1 Probiotic food supplements

A total of 10 commercially available probiotic capsule food supplements available on the Czech market were purchased and analyzed (Supplementary Table A.1). The products were selected based on the following criteria: the label claims specified only total bacterial counts without providing the quantities of individual taxa, and the formulations contained lactobacilli, bifidobacteria, or both groups. Samples were stored for 12 months under ambient (22 °C), refrigeration (6 °C), or freezing (−18 °C) condition in their original packaging, with moisture exposure prevented. For each product, one commercial batch was analyzed in parallel technical replicates (*n* = 3). Lot numbers and expiry dates were recorded prior to analysis. All products were factory-sealed on arrival. An initial analysis was performed immediately upon purchase (T0), followed by subsequent testing after 1 (T1), 3 (T3), 6 (T6), and 12 (T12) months of storage.

### 2.2 Determination of viable bacterial counts

Bifidobacteria, lactobacilli, and total bacterial counts in probiotic products were determined by cultivation. For each analysis, 1 g of sample was obtained by aseptically pooling the contents of multiple emptied capsules and transferred into sterile tubes containing dilution media composed of 5 g/L tryptone, 5 g/L nutrient broth No. 2, and 2.5 g/L yeast extract (all Oxoid, Hampshire, UK), 0.5 g/L L-cysteine, and 1 mL/L Tween 80 (both Sigma-Aldrich, St. Louis, MO, USA). All media were prepared in an oxygen-free carbon dioxide environment as described by Hungate et al. (23) and then sterilized. All samples were homogenized and allowed to stand at ambient temperature for 1 h to rehydrate.

Samples were serially diluted and bacteria determined on semisolid cultivation media as described in Horvathova et al. (24). Briefly: Log₁₀ serial dilutions of the samples were prepared, inoculated onto the Petri dishes, and subsequently overlaid with the corresponding cultivation medium as follows: Wilkins–Chalgren agar (WSP) was supplemented with 5 g/L soya peptone (Oxoid), 0.5 g/L L-cysteine, 1 mL/L Tween 80 (both Sigma-Aldrich) for determination of total bacterial counts, WSP-MUP agar supplemented with 100 mg/L mupirocin (Oxoid) for bifidobacteria, Rogosa agar (Oxoid) with 1.32 mL/L glacial acetic acid (Sigma-Aldrich) for lactobacilli. Total bacterial counts and bifidobacteria were incubated anaerobically at 37 °C for 72 h in anaerobic jars (Anaerobic Plus System; Oxoid), while plates for lactobacilli at 37 °C for 72 h under microaerophilic conditions using a double-layer agar method. After the cultivation, bacterial colonies were counted to determine CFU/g of probiotic product. The limit of detection (LOD) was established at 2 log CFU/g. Additionally, the selectivity of cultivation media was verified by eDT MALDI-TOF MS identification according to Modrackova et al. (25).

#### 2.2.1 Statistical analyses of cultivation data

Statistical processing and data evaluation were conducted using STATISTICA 14 (TIBCO Software Inc., Palo Alto, CA, USA) and MS Excel (Microsoft, Redmond, WA, USA). The normality of the microbial count data (expressed in log CFU/g) was verified using the Shapiro–Wilk W test (α = 0.05). To assess label claim accuracy and stability over time, a one-sample t-test was employed to compare the determined viable counts against the values declared by the manufacturers. These comparisons were performed for each storage condition (ambient, refrigeration, and freezing) across all sampling points from T0 to T12.

Due to the non-normal distribution of data for specific taxa, non-parametric statistical tests were employed. Differences between independent storage conditions, as well as comparisons across all time points independent of storage temperature, were assessed using the Kruskal–Wallis test followed by Dunn’s post-hoc test with Bonferroni correction. Conversely, longitudinal temporal changes within each individual storage condition (repeated measures) were evaluated using the Friedman test, followed by the Wilcoxon signed-rank test with Bonferroni correction for post-hoc pairwise comparisons.

All statistical tests were evaluated at a significance level of α = 0.05. Detailed *p*-values from one-sample t-tests are summarized in Supplementary Tables C.1, C.2, while p- and Z-values from Kruskal–Wallis and Friedman tests and their respective post-hoc analyses are reported in Supplementary Tables C.3–C.6.

## 3 Results

The complete dataset evaluating the quality and stability of the tested probiotic supplements, including the comparison between label claims and long-term storage under varying temperatures, is summarized in Table 1. Baseline assessment of the packaging instructions revealed that the most manufacturers recommended storing the supplements in a dry, dark environment at temperatures up to 25 °C (specifically samples DPS1, DPS2, DPS4, DPS6, DPS7, DPS8, DPS9, DPS10). Product- specific directives varied slightly, with statements such as ‘Store in a dry, dark place at temperatures between 5 °C and 25 °C’ (DPS1, DPS4, DPS6, DPS8, DPS9, DPS10) or ‘Store at ambient temperature between 15 °C and 25 °C’ (DPS7). Notably, one manufacturer explicitly advised keeping the product ideally refrigerated after opening (DPS5). Another manufacturer recommended storage in a cool and dry place, stating it should ideally be kept in the refrigerator (DPS3).

**Table 1:** Changes in total bacterial counts (log CFU/g) in analyzed products during a 12-month storage period under three different temperature conditions.

Sample	Recommended conditions for storage	Declared viable cell count (log CFU/g)	Time	Determined bacterial counts (log CFU/g) ± SD		
				Ambient	Refrigeration	Freezing
DPS1	Dry place at 5–25 °C	10.23	T0	10.41 ± 0.09	-	-
			T1	10.10 ± 0.08	10.36 ± 0.21	10.38 ± 0.15
			T3	**9.94 ± 0.05**	10.33 ± 0.31	10.23 ± 0.20
			T6	**9.75 ± 0.13**	10.29 ± 0.07	10.14 ± 0.18
			T12	**9.48 ± 0.27**	10.20 ± 0.13	9.78 ± 0.19
DPS2	Dry place up to 25 °C	9.80	T0	**8.02 ± 0.11**	-	-
			T1	**7.46 ± 0.28**	**8.01 ± 0.16**	**7.96 ± 0.04**
			T3	**3.87 ± 0.08**	**7.99 ± 0.34**	**7.75 ± 0.13**
			T6	**<2**	**7.98 ± 0.06**	**7.72 ± 0.11**
			T12^exp^	**<2**	**6.96 ± 0.03**	**6.99 ± 0.08**
DPS3	Cool, dry place, preferably refrigerated	10.38	T0	**10.65 ± 0.09**	-	-
			T1	**10.05 ± 0.12**	10.34 ± 0.11	10.39 ± 0.10
			T3	**4.83 ± 0.17**	10.30 ± 0.04	10.38 ± 0.23
			T6	**<2**	**10.14 ± 0.06**	10.36 ± 0.08
			T12^exp^	**<2**	**9.58 ± 0.07**	**10.00 ± 0.10**
DPS4	Dry, dark place at 5–25 °C	10.40	T0	11.05 ± 0.31	-	-
			T1	10.30 ± 0.16	**10.78 ± 0.12**	**10.78 ± 0.04**
			T3	**5.15 ± 0.22**	10.68 ± 0.05	10.59 ± 0.10
			T6	**<2**	10.26 ± 0.26	**10.52 ± 0.03**
			T12	**<2**	**9.46 ± 0.07**	10.51 ± 0.08
DPS5	Ambient, once opened, preferably refrigerated	9.38	T0	9.44 ± 0.08	-	-
			T1	**8.52 ± 0.07**	**8.94 ± 0.15**	9.28 ± 0.28
			T3	**3.95 ± 0.12**	**6.72 ± 0.21**	**9.17 ± 0.08**
			T6	**<2**	**6.23 ± 0.06**	**8.60 ± 0.14**
			T12^exp^	**<2**	**5.98 ± 0.11**	**7.80 ± 0.12**
DPS6	Dry, dark place at 5–25 °C	10.08	T0	**10.63 ± 0.09**	-	-
			T1	**10.47 ± 0.02**	**10.61 ± 0.19**	**10.59 ± 0.08**
			T3	10.46 ± 0.22	**10.53 ± 0.02**	**10.44 ± 0.11**
			T6	10.06 ± 0.24	10.11 ± 0.06	**10.43 ± 0.05**
			T12^exp^	**9.85 ± 0.03**	**8.48 ± 0.06**	9.85 ± 0.48
DPS7	Ambient at 15–25 °C	9.38	T0	**11.47 ± 0.19**	-	-
			T1	9.96 ± 0.35	**10.75 ± 0.04**	**10.63 ± 0.03**
			T3	**7.36 ± 0.01**	**10.73 ± 0.06**	**10.60 ± 0.10**
			T6	**2.97 ± 0.06**	**10.66 ± 0.08**	**10.51 ± 0.01**
			T12	**<2**	**10.43 ± 0.26**	**8.82 ± 0.12**
DPS8	Dry, dark place at 5–25 °C	9.90	T0	**10.98 ± 0.03**	-	-
			T1	**10.79 ± 0.22**	**10.97 ± 0.08**	**10.79 ± 0.22**
			T3	**10.74 ± 0.09**	**10.80 ± 0.13**	**10.73 ± 0.02**
			T6	**10.40 ± 0.19**	**10.72 ± 0.04**	**10.71 ± 0.18**
			T12^exp^	**10.35 ± 0.01**	**10.62 ± 0.10**	**10.69 ± 0.08**
DPS9	Dry, dark place at 5–25 °C	9.90	T0	10.94 ± 0.45	-	-
			T1	**10.35 ± 0.03**	**10.62 ± 0.04**	**10.67 ± 0.04**
			T3	**10.18 ± 0.08**	**10.61 ± 0.06**	**10.62 ± 0.09**
			T6	**10.08 ± 0.02**	**10.52 ± 0.13**	**10.60 ± 0.09**
			T12	**10.02 ± 0.50**	**10.47 ± 0.02**	**10.53 ± 0.20**
DPS10	Dry, dark place at 5–25 °C	9.90	T0	**10.67 ± 0.04**	-	-
			T1	9.84 ± 0.05	10.31 ± 0.21	10.34 ± 0.55
			T3	8.47 ± 0.63	9.83 ± 0.06	10.13 ± 0.19
			T6	**8.35 ± 0.04**	9.81 ± 0.41	9.78 ± 0.13
			T12^exp^	**7.93 ± 0.02**	**9.60 ± 0.11**	9.75 ± 0.07

Time points are defined as follows: T0, baseline (start of storage); T1, 1 month; T3, 3 months; T6, 6 months; and T12, 12 months of storage. Data are presented as means ± standard deviation calculated from technical replicates (*n* = 3). Bold values indicate a statistically significant difference between the observed mean and the manufacturer’s declared log_10_ value, as determined by a one-sample t-test (α = 0.05). The limit of detection (LOD) was established at 2 log CFU/g; results below this threshold were expressed as <2. The dash (−) indicates that at T0, samples had not yet been subjected to specific storage temperatures. The (^exp^) means that the product expired at the time of last measurement.

The initial analysis at T0 showed that 9 of the 10 tested products met the declared CFU counts at the point of purchase. The presence of both target taxa, *Lactobacillaceae* and *Bifidobacterium* spp., and the selectivity of the used media, was verified using eDT MALDI-TOF MS in all analyzed formulations where their presence was declared by the manufacturer.

### 3.1 Assessment of long-term storage impact on viable bacterial counts

Regarding long-term storage stability, it was evaluated whether the products met the manufacturer’s declared microbial counts at the end of their minimum durability period. For this assessment, the final sampling point within the shelf life of each product was utilized. Specifically, for products that reached their expiration date before the conclusion of the study (DPS2, DPS3, DPS5, DPS6, DPS8, DPS10), data from T6 were used for comparison. For remaining samples, compliance was verified at T12. Under these criteria, DPS2 and DPS5 failed to meet the declared counts at the end of their shelf life in all monitored conditions. Out of the 10 products tested, six met the manufacturer’s declared counts at the end of their shelf life under refrigeration condition (DPS1, DPS6, DPS7, DPS8, DPS9, DPS10), six under freezing conditions (DPS3, DPS4, DPS6, DPS8, DPS9, DPS10), and only three at ambient temperature (DPS6, DPS8, DPS9). A notable observation was made for product DPS8, which, although already beyond its minimum durability date at T12, successfully met the declared counts under all three storage conditions. Apart from this instance, the only other product to comply with its label claims after expiration was DPS6, specifically when stored under freezing conditions. In all other cases, the products failed to maintain their declared microbial levels once they surpassed their shelf life in any of the monitored environments.

When evaluating stability relative to manufacturer recommendations, six products (DPS1, DPS2, DPS4, DPS8, DPS9, DPS10) with storage limits up to 25 °C showed optimal counts in refrigerated or freezing conditions, except for DPS6, where counts remained comparable across all three environments. For products specifically suggesting refrigeration, DPS3 (cool, dry, preferably refrigerated) and DPS5 (preferably refrigerated after opening), the counts decreased in the following order: freezing, refrigeration, and ambient condition. A similar trend was observed for DPS7; despite a recommended range of 15–25 °C, refrigerated storage performed better than the other conditions, while ambient storage resulted in the most significant decline in viability.

Statistical analysis focused on identifying the initial point of significant decline relative to the declared values; once a significant reduction was recorded, this trend persisted through all subsequent sampling intervals. Products DPS8 and DPS9 demonstrated exceptional stability throughout the entire 12-month study. No significant deviations from the initial counts (T0) were recorded under any of the three storage conditions (ambient, refrigeration, or freezing) at any sampling interval. In contrast, DPS2 and DPS5 exhibited the lowest stability. For DPS2, a significant decline followed immediately at T0 across all monitored environments. DPS5 showed a similar rapid decline at T1 under ambient and refrigeration, while freezing successfully delayed the significant decline in viability until T3. For the remaining products, the first significant decline in viability was observed to be strictly temperature-dependent, following a trend where stability increased with decreasing temperature. Ambient storage triggered the fastest degradation: significant declines appeared at T1 (DPS3), T3 (DPS1, DPS4, DPS7), T6 (DPS10), and T12 (DPS6). Refrigeration significantly delayed this process, with the first significant reductions recorded at T6 (DPS3) and T12 (DPS4, DPS6, DPS10). Freezing proved most effective, beyond the final sampling point at T12 for DPS3 and DPS7, where a significant decrease in cell numbers was finally observed, all other products in this group remained stable throughout the study. Interestingly, three products (DPS6, DPS8, and DPS9) showed viable counts that significantly above the manufacturer-declared values at T1. While the assessment of label claims identified refrigeration as the most effective method, the statistical analysis showed that freezing was the most effective for maintaining overall stability.

### 3.2 Long-term stability of specific probiotic taxa

Of the 10 products investigated, seven samples containing both bifidobacteria and lactobacilli were selected for taxon-specific analysis (Supplementary Table B.1). Regarding bifidobacteria, in general, a statistically significant decrease in bifidobacterial counts was detected between ambient and refrigeration at T3 time point (Z = 2.43, *p* = 4.48e-02), as well as between ambient T12 (Z = 3.67, *p* = 1.60e-02) and refrigeration T1, and between ambient T12 and freezing T1 (Z = 3.58, *p* = 2.29e-02; Figure 1, Supplementary Table C.4). Although no statistically significant differences were found over time within individual storage conditions for the dependent samples, a decreasing trend in counts was clearly evident (Figure 1, Supplementary Table C.3). Furthermore, considerable variability was observed at the product level. It caused wide spreads of detected bifidobacterial counts with high standard deviations (Supplementary Table B.1).

**Figure 1:**
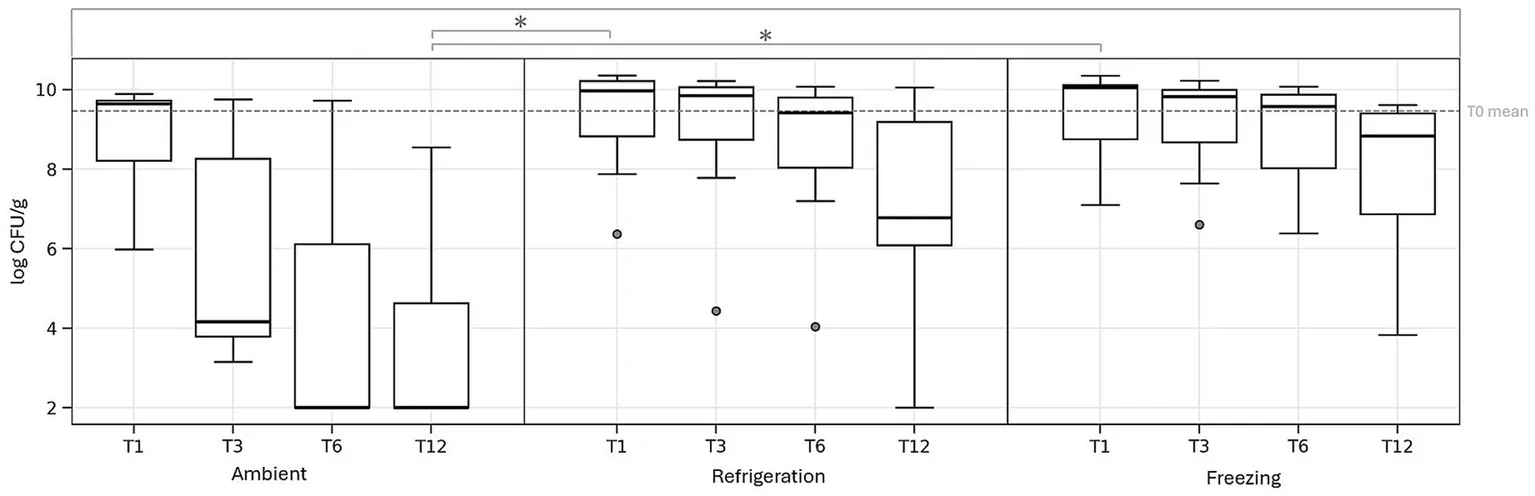
Comparison of bifidobacterial counts (log CFU/g) across storage temperatures at individual time points. Comparison of counts across all time points, independent of storage temperatures, is shown in the upper gray frame. Time points are defined as follows: T0, baseline (start of storage); T1, 1 month; T3, 3 months; T6, 6 months; and T12, 12 months of storage. Boxplots display the median (horizontal line), the interquartile range (box; 25th–75th percentile), and the whiskers representing the most extreme values within 1.5 × the interquartile range; individual points denote outliers. The dashed horizontal line represents the mean initial bifidobacterial count at T0 (9.46 log CFU/g), which was used as a reference value due to the absence of taxon-specific viable counts on product labels. Horizontal brackets with asterisks (*) denote statistically significant differences between independent samples, as determined by the Kruskal–Wallis test followed by Dunn’s post-hoc test with Bonferroni correction (*p* < 0.05). Longitudinal changes within individual storage conditions were assessed using the Friedman test, yielding no statistically significant pairwise differences.

In more detail (Supplementary Table B.1), ambient storage induced the most rapid reduction in viability, with significant declines initiated at T1 for products DPS2, DPS5, DPS6, and DPS7 compared to T0 representing baseline reference due to the absence of taxon-specific counts on product labels, and at T3 for DPS1, DPS3, and DPS4. Refrigeration successfully delayed the first significant decline until T6 for products DPS1, DPS2, DPS3, and DPS6, and until T12 for DPS7, whereas DPS5 exhibited high sensitivity with significant losses already at T1. In the freezing condition, stability was maintained until T6 for DPS1, DPS2, and DPS3, while significant reductions were recorded at T3 for the remaining products, specifically DPS4, DPS5, DPS6, and DPS7.

For lactobacilli, comparisons across all time points, independent of storage temperatures, showed no statistically significant differences (Figure 2, Supplementary Table C.6). However, a consistent decreasing trend in viable lactobacilli over time was evident (Supplementary Table C.5), and similar to the bifidobacterial group, pronounced variability at the individual product level resulted in a wide range of detected lactobacilli abundances and high standard deviations (Supplementary Table B.1).

**Figure 2:**
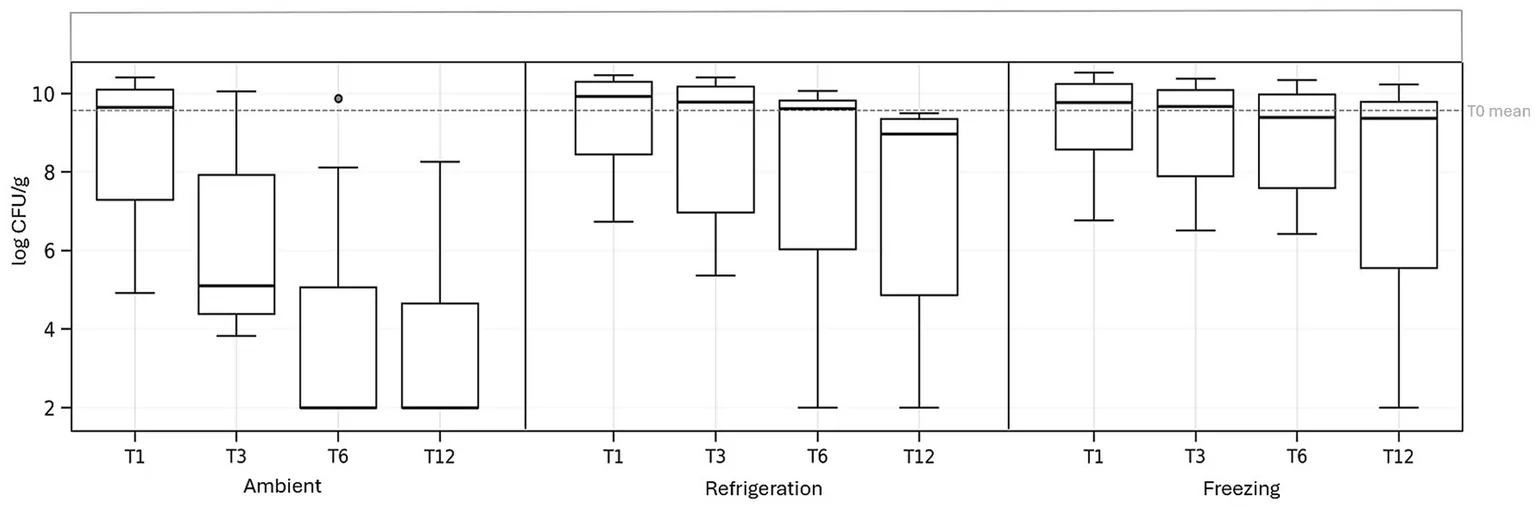
Comparison of lactobacilli counts (log CFU/g) across storage temperatures at individual time points. Comparison of counts across all time points, independent of storage temperatures, is shown in the upper gray frame. Time points are defined as follows: T0, baseline (start of storage); T1, 1 month; T3, 3 months; T6, 6 months; and T12, 12 months of storage. Boxplots display the median (horizontal line), the interquartile range (box; 25th–75th percentile), and the whiskers representing the most extreme values within 1.5 × the interquartile range; individual points denote outliers. The dashed horizontal line represents the mean initial lactobacilli count at T0 (9.57 log CFU/g), which was used as a reference value due to the absence of taxon-specific viable counts on product labels. Differences between independent samples and longitudinal changes within individual storage conditions were assessed using the Kruskal-Wallis and Friedman tests, respectively. Neither analysis yielded statistically significant differences (*p* > 0.05).

In more detail, ambient storage led to significant declines at T1 for DPS2, DPS5, DPS6, and DPS7, and at T3 for DPS1, DPS3, and DPS4. Under refrigeration conditions, significant declines were recorded at T1 for DPS2, DPS5, DPS6, and DPS7, while the decline was delayed until T3 for DPS4 and T6 for DPS1 and DPS3. Freezing condition failed to maintain stability beyond T1 for DPS6 and DPS7, whereas the initial point of significant decline was recorded at T3 for products DPS1, DPS3, DPS4, and DPS5, and at T6 for DPS2.

Notably, for both taxa in products DPS1 and DPS3, refrigeration proved more effective than freezing in delaying the first significant loss of viability. Regarding taxon-specific stability, it is not possible to unequivocally determine whether refrigeration or freezing was superior; however, it is evident that ambient storage represents the least effective condition for maintaining viability. The results further showed that in several instances, the total probiotic counts met the determined values primarily due to the stability of only one of the monitored taxa. This phenomenon was observed in products DPS5 and DPS7. In product DPS5, the final viability at T12 under refrigeration was sustained exclusively by lactobacilli (5.96 ± 0.04 log CFU/g, a value nearly identical to the total bacterial counts recorded at this interval), while the bifidobacteria fell below the detection limit (<2 log CFU/g). Conversely, a reverse trend was observed in DPS7 (at T12 under refrigeration), where lactobacilli proved highly unstable (<2 log CFU/g), whereas bifidobacteria reached 10.06 ± 0.02 log CFU/g.

### 3.3 Taxonomic nomenclature and strain-level labelling

Across the 10 analyzed probiotic samples, none of the products adhered to updated taxonomic nomenclature reflecting the reclassification of the former *Lactobacillus* genus (52). Specifically, labels continued to use legacy species designations such as “*Lactobacillus casei*,” “*Lactobacillus rhamnosus*,” “*Lactobacillus plantarum*,” and “*Lactobacillus paracasei*,” rather than the corresponding updated genera (*Lacticaseibacillus casei, Lacticaseibacillus rhamnosus, Lactiplantibacillus plantarum*, and *Lacticaseibacillus paracasei*).

Incomplete taxonomic resolution was also observed within the genus *Bifidobacterium*. In DPS2, DPS5, and DPS6, *Bifidobacterium longum* was declared without subspecies information. In addition, products declaring *Bifidobacterium animalis* subsp. *lactis* (DPS1, DPS3, and DPS4) listed this taxon only as *B. animalis*, omitting the subspecies designation.

Strain-level identification on product labels was provided for 4 out of 10 supplements (DPS1, DPS4, DPS5, and DPS6), whereas the remaining products reported taxa only at the species level.

## 4 Discussion

Probiotic supplement quality is commonly assessed through label-declared viable counts, yet standardized evaluation frameworks and binding storage specifications remain limited. In the absence of clear guidance on approaches for verifying viability and microbial counts, culture-based CFU enumeration remains the reference method and was therefore applied in this study. We performed 12-month longitudinal testing of probiotic products stored under ambient, refrigeration and freezing conditions. Refrigeration best preserved manufacturer-declared counts overall. However, taxon-specific stability did not clearly differentiate refrigeration from freezing, precluding an unequivocal ranking between these two conditions. Ambient temperature was the least effective for maintaining the viability of probiotics. This pattern is consistent with prior studies showing superior stability under cold conditions (26–29). Specifically, Bruno and Shah (30) demonstrated that maintaining a temperature of −18 °C is optimal for the long-term storage of lyophilized probiotics to maximize bifidobacteria viability, whereas storage at 20 °C is unsuitable, leading to a significant reduction in viable bacterial counts. However, the limitation of previous studies is their focus on single-strain probiotics. By contrast, we analyzed multi-strain probiotic formulations, in which stability may reflect more complex dynamics arising from co-occurring taxa with distinct sensitivities and potential interspecies effects within a shared matrix (31). In the commercial landscape, a discrepancy exists between evidence-based recommendations and prevailing marketing strategies. Clinicians often recommend single-strain or narrowly defined 2–3 strain formulations to support strain specificity and reproducibility (32, 33), yet the market is increasingly dominated by multi-strain products (34). These are frequently marketed as synergistic combinations, but experimental evidence supporting the stability and functional compatibility of complex consortia still remains limited (35, 36).

Substances, such as fermentable fibers or prebiotic oligosaccharides (e.g., inulin, FOS, GOS, resistant starch) are added to probiotic preparations not only to preserve viability but also to enhance survival during gastrointestinal transit of the product. Such synbiotic combinations are intended to support microbial persistence and metabolic activity (37). However, at the same time, some of these substances can complicate long-term storage by increasing moisture uptake due to hygroscopicity (38). In general, protectants are widely used in freeze-dried and solid-state formulations (8). Common strategies include disaccharides (trehalose, sucrose) as cryo- and lyo-protectants that stabilize membranes and proteins via water replacement and vitrification (10), carbohydrate carriers (maltodextrin, lactose) as bulking agents supporting matrix formation, and protein-based systems (skim milk powder, whey protein) providing a protective protein–lactose matrix that improves survival during dehydration and rehydration stress (10, 39). Furthermore, inulin with a prebiotic functionality, may also act as stabilizing matrices by forming glassy structures that immobilize cells and reduce molecular mobility (9). More recently, hydrocolloid and polymer-based systems such as hydroxypropyl methylcellulose (HPMC) have been explored to improve structural integrity and reduce moisture-driven destabilization (40). Interestingly, DPS10 showed the poorest stability and was the only product encapsulated in gelatin rather than HPMC, while DPS8 and DPS9 from the same manufacturer exhibited markedly better stability. While this observation does not establish causality, it is consistent with the notion that capsule material may contribute to moisture-related stability differences. Collectively, these excipients can increase stability by lowering water activity, limiting membrane/protein damage, and reducing structural collapse during processing and storage (8). Although the effect of the individual contained protectants cannot be inferred, it is interesting that, DPS1, among products containing both taxa, uniquely combining maltodextrin and inulin, maintained among the highest viable counts after 12 months of storage. These results suggest that combining bulking agents with prebiotic carbohydrates may enhance long-term probiotic stability. Nevertheless, further studies are required to confirm this potential synergistic effect and clarify the underlying mechanisms.

Across products, species composition was consistently linked to bifidobacterial survival. Products lacking *Bifidobacterium animalis* subsp. *lactis* (DPS2, DPS5, and DPS6) generally showed lower bifidobacterial counts throughout storage than formulations containing this subspecies. This aligns with previous report describing differences in technological robustness among *Bifidobacterium* spp. (41). The comparatively high stability of *B. animalis* subsp. *lactis* is consistent with its technological robustness, including improved tolerance to oxidative stress and acidic conditions and better survival during processing and storage than other bifidobacterial species (42–44). This likely contributes to its frequent use where stability is a primary requirement. From a physiological perspective, however, it remains relevant to question whether highly robust, technologically adapted strains fully reflect host-associated features of the human gut microbiota. *B. animalis* subsp. *lactis* is not typically considered a dominant core gut member, unlike *B. longum*, *B. adolescentis*, or *B. breve*, which are more consistently associated with intestinal colonization and persistence (45). Within the family *Lactobacillaceae*, species such as *Lacticaseibacillus rhamnosus*, *Lactiplantibacillus plantarum*, and *Limosilactobacillus reuteri* are frequently detected in the gastrointestinal tract, yet evidence suggests many gut lactobacilli represent transient populations shaped by diet and intake of fermented foods or supplements rather than stable autochthonous colonizers (46, 47). In this context, it remains an open question whether the use of highly technologically robust but potentially less autochthonous strains represents an optimal strategy for all probiotic applications, or whether formulation strategies should more explicitly consider host-associated origin and adaptation alongside industrial stability requirements.

Most of the products did not maintain label-declared viable counts over time. While 9/10 products met declared counts at baseline (T0), compliance decreased toward the end of minimum durability. Under ambient storage, only 3/10 products remained compliant at the final time point; refrigeration and freezing improved outcomes to 6/10 compliant products. Notably, 2/10 products failed to meet declared counts under any condition, even before expiration. This suggests that viability loss in these cases may reflect factors beyond storage temperature alone, such as formulation-intrinsic properties or insufficient overage. Overage is widely used in probiotic manufacturing to compensate for anticipated viability losses over shelf life (48). In our dataset, DPS7 showed the largest overage (approximately two orders of magnitude at baseline), whereas DPS8 and DPS9 showed overages of one order of magnitude. Divergence between declared and measured counts has been repeatedly reported: Marinova et al. (12) found none of the 16 tested probiotic supplements fully met label claims and detected no viable bacteria in some samples; Toscano et al. (49) reported noncompliance in 10/24 products, including several with no detectable viable cells; and Mohar et al. (13) similarly reported frequent shortfalls, with 12% of tested supplements containing insufficient viable bacteria. Discrepancies between declared and detected species have also been described, particularly in multi-strain formulations.

Finally, taxonomic inconsistency and outdated nomenclature persist, including within the scientific literature. Hill et al. (50) noted that incomplete or incorrect taxonomic designations remain common in probiotic publications. Han et al. (51) provides an illustrative example, referring to “*Lactobacillus subtilis*” and “*Streptococcus faecium*.” The latter reflects outdated nomenclature and most likely corresponds to *Enterococcus faecium*, while “*Lactobacillus subtilis*” does not represent a valid taxonomic entity and likely reflects misidentification of *Bacillus subtilis*. In addition, many manufacturers have not adopted the updated *Lactobacillus* genus taxonomy after reclassification by Zheng et al. (52). This was evident in our sample set. Taxonomic nomenclature and label resolution were frequently inconsistent across products, including incomplete subspecies information and limited strain-level identification. Given that probiotic effects are strain-specific (3), such labeling gaps hinder both functional interpretation and clinical applicability.

An interesting finding in our study was the temporal lag between the point when a product failed to meet its label claim and the point of a statistically significant decline in viability. In several cases, the total counts showed no significant decline compared to the label-declared counts, even though they had numerically fallen below the declared threshold. One such example is sample DPS1 stored at ambient temperature, while it failed to meet the label claim as early as T1, a statistically significant decrease was not observed until T3. This discrepancy raises critical questions regarding the criteria for regulatory compliance, should a product be deemed non-compliant immediately upon falling below the nominal label value, or only once a statistically significant deviation from the declaration is established? This challenge in quality assessment is reflected in the guidelines of the Italian Ministry of Health. According to IPA Europe (53) Italy represents one of the five largest probiotic markets within the EU. As specified in the Italian Ministry of Health guidelines on probiotics and prebiotics (54), the number of cells present in a probiotic product must be listed on the label. Moreover, this amount must be guaranteed until the end of the product shelf-life at the specified storage conditions, with an uncertainty of 0.5 log. The adoption of such factor acknowledges the technical limitations of CFU quantification and the natural fluctuations in microbial counts. Incorporating a standardized tolerance limit into international regulations could provide a more scientifically grounded framework for quality control.

## 5 Conclusion

Overall, our findings demonstrate that storage temperature remains the crucial determinant of probiotic viability, with ambient conditions leading to the greatest losses and cold storage substantially improving long-term stability. Products containing *B. animalis* subsp. *lactis* generally exhibited better bifidobacterial stability than formulations lacking this taxon. Products combining bulking agents with prebiotic carbohydrates, such as maltodextrin and inulin, or formulated in moisture-protective HPMC capsules, may have contributed to improved resistance to storage-related viability loss. Collectively, these results suggest that probiotic formulation should be designed in close alignment with intended storage conditions. Strain selection, excipient composition, encapsulation strategy, and storage requirements should be considered as an integrated system to ensure sustained viability and consistent product quality throughout shelf life.

## Data Availability

The original contributions presented in the study are included in the article/Supplementary material, further inquiries can be directed to the corresponding author.
