# Supplementary material for: Storage temperature as a determinant of probiotic long-term stability in commercial dietary supplements
Source: Front Nutr. 2026 Sep 17;13:1933900. doi: 10.3389/fnut.2026.1933900 (PMC13627402; doi:10.3389/fnut.2026.1933900)
Supplement: Supplementary file 1 [file Supplementary_file_1.zip › Supplementary Table B.docx]

**Supplementary Table B.1:** Changes in total bifidobacteria and lactobacilli counts (log CFU/g) in analysed products during a 12-month storage period under three different temperature conditions.

Time points are defined as follows: T0, baseline (start of storage); T1, 1 month; T3, 3 months; T6, 6 months; and T12, 12 months of storage. Bold values indicate a statistically significant difference between the observed mean and the manufacturer's declared log10 value, as determined by a one-sample t-test (α = 0.05). The limit of detection (LOD) was established at 2 log CFU/g, results below this threshold were expressed as <2. The dash (-) indicates that at T0, samples had not yet been subjected to specific storage temperatures. The (^exp^) mean that the product expired at the time of last measurement.

| **Sample** | **Time** | **Determined BIF counts**  **(log CFU/g) ± SD** | | | **Determined LAB counts**  **(log CFU/g) ± SD** | | |
| --- | --- | --- | --- | --- | --- | --- | --- |
|  |  | **Ambient** | **Refrigeration** | **Freezing** | **Ambient** | **Refrigeration** | **Freezing** |
| **DPS1** | T0 | 10.37 ± 0.09 | - | - | 10.13 ± 0.40 | - | - |
|  | T1 | 9.90 ± 0.26 | 10.20 ± 0.14 | 10.35 ± 0.21 | 9.65 ± 0.31 | 9.93 ± 0.13 | 9.77 ± 0.72 |
|  | T3 | **9.75 ± 0.11** | 10.18 ± 0.09 | 10.23 ± 0.13 | **9.64 ± 0.03** | 9.78 ± 0.19 | **9.67 ± 0.08** |
|  | T6 | **9.73 ± 0.13** | **9.89 ± 0.09** | **10.07 ± 0.06** | **8.11 ± 0.08** | **9.62 ± 0.06** | **9.39 ± 0.16** |
|  | T12 | **7.26 ± 0.18** | **9.82 ± 0.04** | **9.61 ± 0.15** | **7.30 ± 0.07** | **9.27 ± 0.03** | **9.37 ± 0.06** |
| **DPS2** | T0 | 7.91 ± 0.15 | - | - | 7.03 ± 0.11 | - | - |
|  | T1 | **6.85 ± 0.04** | 7.88 ± 0.23 | 7.77 ± 0.12 | **6.20 ± 0.09** | **6.73 ± 0.11** | 6.77 ± 0.15 |
|  | T3 | **3.87 ± 0.08** | 7.78 ± 0.22 | 7.64 ± 0.15 | **3.05 ± 0.02** | **5.51 ± 0.05** | 6.51 ± 0.24 |
|  | T6 | **<2** | **7.20 ± 0.09** | **7.59 ± 0.03** | **<2** | **5.16 ± 0.18** | 6.48 ± 0.37 |
|  | T12^exp^ | **<2** | **6.19 ± 0.17** | **5.77 ± 0.40** | **<2** | **3.76 ± 0.08** | **3.84 ± 0.06** |
| **DPS3** | T0 | 9.81 ± 0.09 | - | - | 10.29 ± 0.19 | - | - |
|  | T1 | 9.58 ± 0.28 | 9.78 ± 0.18 | 9.74 ± 0.03 | 10.01 ± 0.15 | 10.26 ± 0.19 | 10.17 ± 0.09 |
|  | T3 | **3.72 ± 0.21** | 9.71 ± 0.16 | 9.71 ± 0.10 | **4.81 ± 0.12** | 10.22 ± 0.06 | **9.91 ± 0.05** |
|  | T6 | **<2** | **9.41 ± 0.10** | **9.57 ± 0.03** | **<2** | **9.98 ± 0.09** | **9.81 ± 0.19** |
|  | T12^exp^ | **<2** | **8.55 ± 0.06** | **8.83 ± 0.07** | **<2** | **9.43 ± 0.05** | **9.69 ± 0.14** |
| **DPS4** | T0 | 10.36 ± 0.10 | - | - | 10.71 ± 0.36 | - | - |
|  | T1 | 9.65 ± 0.63 | 10.36 ± 0.21 | 10.15 ± 0.32 | 10.41 ± 0.15 | 10.46 ± 0.13 | 10.53 ± 0.14 |
|  | T3 | **4.16 ± 0.12** | **9.95 ± 0.11** | **10.09 ± 0.08** | **5.11 ± 0.11** | **10.41 ± 0.07** | **10.38 ± 0.06** |
|  | T6 | **<2** | **9.71 ± 0.06** | **10.05 ± 0.05** | **<2** | **10.06 ± 0.05** | **10.34 ± 0.03** |
|  | T12 | **<2** | **6.77 ± 0.29** | **9.33 ± 0.12** | **<2** | **8.97 ± 0.05** | **10.23 ± 0.11** |
| **DPS5** | T0 | 7.30 ± 0.18 | - | - | 9.32 ± 0.13 | - | - |
|  | T1 | **5.98 ± 0.02** | **6.37 ± 0.06** | 7.10 ± 0.09 | **8.38 ± 0.09** | **8.56 ± 0.12** | 8.88 ± 0.37 |
|  | T3 | **3.15 ± 0.11** | **4.43 ± 0.57** | **6.60 ± 0.21** | **3.95 ± 0.12** | **8.43 ± 0.29** | **8.72 ± 0.05** |
|  | T6 | **<2** | **4.04 ± 0.22** | **6.39 ± 0.33** | **<2** | **6.89 ± 0.16** | **8.69 ± 0.19** |
|  | T12^exp^ | **<2** | **<2** | **3.83 ± 0.87** | **<2** | **5.96 ± 0.04** | **7.25 ± 0.10** |
| **DPS6** | T0 | 10.08 ± 0.11 | - | - | 10.84 ± 0.06 | - | - |
|  | T1 | **9.64 ± 0.04** | 9.97 ± 0.08 | 10.05 ± 0.03 | **10.18 ± 0.09** | **10.33 ± 0.10** | **10.32 ± 0.08** |
|  | T3 | **9.37 ± 0.13** | 9.85 ± 0.26 | **9.82 ± 0.07** | **10.05 ± 0.14** | **10.12 ± 0.08** | **10.27 ± 0.05** |
|  | T6 | **9.31 ± 0.26** | **8.88 ± 0.21** | **9.70 ± 0.08** | **9.87 ± 0.03** | **9.66 ± 0.05** | **10.15 ± 0.09** |
|  | T12^exp^ | **8.55 ± 0.01** | **5.98 ± 0.14** | **9.48 ± 0.04** | **8.26 ± 0.01** | **9.50 ± 0.14** | **9.87 ± 0.11** |
| **DPS7** | T0 | 10.42 ± 0.12 | - | - | 8.67 ± 0.20 | - | - |
|  | T1 | **9.81 ± 0.04** | 10.24 ± 0.28 | 10.07 ± 0.15 | **4.92 ± 0.14** | **8.33 ± 0.02** | **8.27 ± 0.03** |
|  | T3 | **7.16 ± 0.11** | 10.22 ± 0.12 | **9.91 ± 0.03** | **3.82 ± 0.18** | **5.36 ± 0.14** | **7.06 ± 0.26** |
|  | T6 | **2.92 ± 0.06** | 10.07 ± 0.24 | **8.46 ± 0.04** | **<2** | **<2** | **6.42 ± 0.03** |
|  | T12 | **<2** | **10.06 ± 0.02** | **7.96 ± 0.16** | **<2** | **<2** | **<2** |
