# Supplementary material for: Storage temperature as a determinant of probiotic long-term stability in commercial dietary supplements
Source: Front Nutr. 2026 Sep 17;13:1933900. doi: 10.3389/fnut.2026.1933900 (PMC13627402; doi:10.3389/fnut.2026.1933900)
Supplement: Supplementary file 1 [file Supplementary_file_1.zip › Supplementary Table A.docx]

**Supplementary Table A.1:** Overview and characteristics of the analysed probiotic supplements (Information taken directly from the manufacturer's packaging)

The supplements were supplied in capsule (a solid dosage form with a hard or soft shell of various shapes and capacities, enclosing the active ingredient(s) and excipients) dosage form. Presented definition is in accordance with the general monographs of the European Pharmacopoeia (Ph. Eur. 12th Ed.). MDD, Minimum Durability Date; CFU, colony forming unit.

| Code | MDD | Declared probiotic strains | Other ingredients | Recommended storage conditions | Declared CFU/g |
| --- | --- | --- | --- | --- | --- |
| DPS1 | 01/2026 | *Bifidobacterium lactis* BL2*, Lactobacillus acidophilus* LH5*, Lactobacillus casei* LC1*, Bifidobacterium bifidum* BF2 | IMG® (complex of biologically active polysaccharides based on pleuran from *Pleurotus ostreatus*), maltodextrin (bulking agent), fructooligosaccharides, inulin, corn starch (bulking agent), magnesium salts of fatty acids (anti-caking agent), silicon dioxide (anti-caking agent), cholecalciferol (vitamin D), capsule shell (hydroxypropyl methylcellulose) | Store in a dry place at a temperature between 5 °C and 25 °C | 1.7 x 10^10^ |
| DPS2 | 03/2025 | *Lactobacillus plantarum, Lactobacillus rhamnosus, Lactobacillus acidophilus* LA-14*, Bifidobacterium bifidum, Bifidobacterium longum, Lactobacillus casei* | Inulin, sweeteners (fructose, sorbitol, sucralose), cane sugar, flavouring (cranberry + raspberry), colour (beetroot powder [betanin]), malted powder (barley, wheat), fructooligosaccharides, citric acid, capsule shell (hydroxypropyl methylcellulose) | Store in a dry, dark place at temperatures up to 25 °C | 6.3x 10^9^ |
| DPS3 | 02/2025 | *Lactobacillus paracasei, Lactobacillus acidophilus, Lactobacillus rhamnosus, Bifidobacterium lactis, Bifidobacterium breve, Lactobacillus plantarum* | Maltodextrin, L-ascorbic acid, capsule shell (hydroxypropyl methylcellulose) | Store in a cool, dry place, preferably refrigerated | 2.4 x 10^10^ |
| DPS4 | 07/2025 | *Lactobacillus acidophilus* LA85*, Bifidobacterium lactis* BLa80*, Lactobacillus rhamnosus* LRa05*, Bifidobacterium breve* BBr60*, Lactobacillus bulgaricus* LB42*, Lactobacillus plantarum* Lp90*, Lactobacillus casei* LC89*, Bifidobacterium bifidum* BBi32*, Lactobacillus paracasei* LC86 | Inulin, L-ascorbic acid, capsule shell (hydroxypropyl methylcellulose) | Store in a dry, dark place at temperatures between 5 °C and 25 °C | 2.5 x 10^10^ |
| DPS5 | 03/2025 | *Lactobacillus rhamnosus* HA-111*, Bifidobacteium longum* HA-135*, Lactobacillus acidophilus* HA-122*, Bifidobacteium bifidum* HA-132*, Lactobacillus reuteri* HA-188 | Sucrose, maltodextrin, sweetener (sorbitol), cherry flavouring, thickener (microcrystalline cellulose), anti-caking agent (magnesium stearate), acidity regulator (citric acid), antioxidant (ascorbic acid), capsule shell (hydroxypropyl methylcellulose) | Store at ambient temperature, once opened, preferably keep refrigerated | 2.4 x 10^9^ |
| DPS6 | 03/2025 | *Lactobacillus helveticus* R0052R*, Bifidobacterium longum* R0175R*, Lactobacillus ssp. lactis* R1058R*, Bifidobacterium breve* R0070R*, Lactobacillus rhamnosus* R0011R*, Bifidobacterium bifidum* R0071R | Potato starch, anti-caking agent (magnesium salts of fatty acids), antioxidant (L-ascorbic acid), capsule shell (hydroxypropyl methylcellulose) | Store in a dry, dark place at temperatures between 5 °C and 25 °C | 1.2 x 10^10^ |
| DPS7 | 06/2025 | *Lactobacillus acidophilus, Bifidobacterium animalis lactis* | Dextrose, anti-caking agent (magnesium salts of fatty acids), colour (calcium carbonate), capsule shell (hydroxypropyl methylcellulose) | Store at ambient temperature between 15 °C and 25 °C | 2.4 x 10^9^ |
| DPS8 | 02/2025 | *Lactobacillus acidophilus*, *Lacticaseibacillus rhamnosus* | Maltodextrin (bulking agent), magnesium stearate (anti-caking agent), ascorbic acid (antioxidant), capsule shell (hydroxypropyl methylcellulose) | Store in a dry, dark place at temperatures between 5 °C and 25 °C | 8.0 x 10^9^ |
| DPS9 | 01/2026 | *Lacticaseibacillus rhamnosus*, *Lactobacillus acidophilus* | Lactose, dried whey, maltodextrin (bulking agent), magnesium stearate (anti-caking agent), ascorbic acid (antioxidant), capsule shell (hydroxypropyl methylcellulose) | Store in a dry, dark place at temperatures between 5 °C and 25 °C | 8.0 x 10^9^ |
| DPS10 | 03/2025 | *Lactobacillus helveticus, Lactobacillus rhamnosus* | Maltodextrin, magnesium stearate, ascorbic acid, capsule shell (gelatine) | Store in a dry, dark place at temperatures between 5 °C and 25 °C | 8.0 x 10^9^ |
