# Supplementary material for: Storage temperature as a determinant of probiotic long-term stability in commercial dietary supplements
Source: Front Nutr. 2026 Sep 17;13:1933900. doi: 10.3389/fnut.2026.1933900 (PMC13627402; doi:10.3389/fnut.2026.1933900)
Supplement: Supplementary file 1 [file Supplementary_file_1.zip › Supplementary Table C.docx]

**Supplementary Table C.1:** P-values in one-sample t-test (Changes in total bacterial counts)

Time points are defined as follows: T0, baseline (start of storage); T1, 1 month; T3, 3 months; T6, 6 months; and T12, 12 months of storage. Bold values indicate a statistically significant difference between the observed mean and the manufacturer's declared log10 value, as determined by a one-sample t-test (α = 0.05). n.t. – not tested (All CFU/g values were below the limit of detection (LOD), resulting in a standard deviation of zero and precluding the application of a t-test. However, consistent with the statistically significant decrease observed in preceding measurements, this result was interpreted as a continuation of the declining trend.). The dash (-) indicates that at T0, samples had not yet been subjected to specific storage temperatures. The (^exp^) mean that the product expired at the time of last measurement.

| **Sample** | **Time** | **Determined bacterial counts**  **(log CFU/g) ± SD** | | |
| --- | --- | --- | --- | --- |
|  |  | **Ambient** | **Refrigeration** | **Freezing** |
| **DPS1** | T0 | 7.42e-2 | - | - |
|  | T1 | 0.11 | 0.40 | 0.23 |
|  | T3 | **9.76e-3** | 0.63 | 1.00 |
|  | T6 | **2.36e-2** | 0.28 | 0.48 |
|  | T12 | **4.06e-2** | 0.73 | 5.46e-2 |
| **DPS2** | T0 | **1.27e-3** | - | - |
|  | T1 | **4.74e-3** | **2.65e-3** | **1.57e-4** |
|  | T3 | **0.61e-4** | **1.16e-2** | **1.34e-3** |
|  | T6 | **n.t.** | **3.62e-4** | **9.31e-4** |
|  | T12 ^exp^ | **n.t.** | **0.37e-4** | **2.70e-4** |
| **DPS3** | T0 | **3.51e-2** | - | - |
|  | T1 | **4.14e-2** | 0.59 | 0.88 |
|  | T3 | **3.13e-4** | 7.41e-2 | 1.00 |
|  | T6 | **n.t.** | **2.02e-2** | 0.71 |
|  | T12 ^exp^ | **n.t.** | **2.54e-3** | **2.23e-2** |
| **DPS4** | T0 | 6.82e-2 | - | - |
|  | T1 | 0.39 | **3.17e-2** | **3.67e-3** |
|  | T3 | **5.85e-4** | 1.05e-2 | 8.12e-2 |
|  | T6 | **n.t.** | 0.44 | **2.02e-2** |
|  | T12 | **n.t.** | **1.84e-3** | 0.14 |
| **DPS5** | T0 | 0.32 | - | - |
|  | T1 | **2.20e-3** | **3.66e-2** | 0.60 |
|  | T3 | **1.63e-4** | **2.07e-3** | **4.51e-2** |
|  | T6 | **n.t.** | **1.21e-4** | **1.06e-2** |
|  | T12 ^exp^ | **n.t.** | **1.39e-4** | **1.92e-3** |
| **DPS6** | T0 | **8.81e-3** | - | - |
|  | T1 | **8.75e-4** | **4.03e-2** | **8.10e-3** |
|  | T3 | 9.59e-2 | **6.58e-4** | **2.97e-2** |
|  | T6 | 0.90 | 0.48 | **6.73e-3** |
|  | T12 ^exp^ | **5.62e-3** | **4.68e-4** | 0.49 |
| **DPS7** | T0 | **2.74e-3** | - | - |
|  | T1 | 0.10 | **2.84e-4** | **1.92e-4** |
|  | T3 | **0.08e-4** | **6.58e-4** | **2.23e-3** |
|  | T6 | **0.29e-4** | **1.30e-3** | **0.26e-4** |
|  | T12 | **n.t.** | **1.98e-2** | **1.50e-2** |
| **DPS8** | T0 | **2.57e-4** | - | - |
|  | T1 | **1.98e-2** | **1.86e-3** | **1.98e-2** |
|  | T3 | **3.81e-3** | **6.88e-3** | **1.93e-4** |
|  | T6 | **4.49e-2** | **7.92e-4** | **1.61e-2** |
|  | T12 ^exp^ | **1.65e-4** | **6.37e-4-3** | **3.40e-3** |
| **DPS9** | T0 | 5.71e-2 | - | - |
|  | T1 | **1.48e-3** | **1.03e-3** | **8.98e-4** |
|  | T3 | **2.61e-2** | **2.37e-3** | **5.17e-3** |
|  | T6 | **4.09e-3** | **1.43e-2** | **5.47e-3** |
|  | T12 | 0.72 | **4.10e-4** | **3.20e-3** |
| **DPS10** | T0 | **8.98e-4** | - | - |
|  | T1 | 0.17 | 7.74e-2 | 0.30 |
|  | T3 | 5.90e-2 | 0.18 | 0.17 |
|  | T6 | **2.22e-4** | 0.74 | 0.25 |
|  | T12 ^exp^ | **0.34e-4** | **4.20e-2** | 6.55e-2 |

**Supplementary Table C.2:** P-values in one-sample t-test (Changes in total bifidobacteria and lactobacilli counts) Time points are defined as follows: T1, 1 month; T3, 3 months; T6, 6 months; and T12, 12 months of storage. Bold values indicate a statistically significant difference between the observed mean and T0, which served as the baseline reference due to the absence of taxon‑specific viable counts on product labels., as determined by a one-sample t-test (α = 0.05). n.t. – not tested (All CFU/g values were below the limit of detection (LOD), resulting in a standard deviation of zero and precluding the application of a t-test. However, consistent with the statistically significant decrease observed in preceding measurements, this result was interpreted as a continuation of the declining trend.). The (^exp^) mean that the product expired at the time of last measurement.

| **Sample** | **Time** | **Determined BIF counts**  **(log CFU/g) ± SD** | | | **Determined LAB counts**  **(log CFU/g) ± SD** | | |
| --- | --- | --- | --- | --- | --- | --- | --- |
|  |  | **Ambient** | **Refrigeration** | **Freezing** | **Ambient** | **Refrigeration** | **Freezing** |
| **DPS1** | T1 | 8.87e-2 | 0.17 | 0.88 | 0.12 | 0.12 | 0.47 |
|  | T3 | **1.03e-2** | 6.73e-2 | 0.20 | **1.25e-3** | **8.58e-2** | **9.93e-3** |
|  | T6 | **1.35e-2** | **1.15e-2** | **1.31e-2** | **5.22e-4** | **4.58e-3** | **1.52e-2** |
|  | T12 | **1.12e-3** | **1.76e-3** | **1.27e-2** | **2.04e-4** | **4.05e-4** | **2.07e-3** |
| **DPS2** | T1 | **4.74e-4** | 0.84 | 0.18 | **3.90e-3** | **4.20e-2** | 9.53e-2 |
|  | T3 | **1.31e-4** | 0.41 | 8.93e-2 | **0.08e-4** | **3.55e-2** | 6.42e-2 |
|  | T6 | **n.t.** | **5.31e-3** | **2.92e-3** | **n.t.** | **3.07e-3** | 0.12 |
|  | T12 ^exp^ | **n.t.** | **3.24e-3** | **1.14e-2** | **n.t.** | **1.76e-4** | **1.18e-4** |
| **DPS3** | T1 | 0.29 | 0.80 | 5.61e-2 | 8.38e-2 | 0.81 | 0.15 |
|  | T3 | **3.96e-4** | 0.39 | 0.23 | **1.60e-4** | 0.18 | **5.72e-3** |
|  | T6 | **n.t.** | **2.02e-2** | **5.17e-3** | **n.t.** | **2.70e-2** | **4.85e-2** |
|  | T12 ^exp^ | **n.t.** | **7.55e-4** | **1.70e-3** | **n.t.** | **1.13e-3** | **1.77e-2** |
| **DPS4** | T1 | 0.19 | 1.00 | 0.37 | 7.42e-2 | 7.95e-2 | 0.16 |
|  | T3 | **1.25e-4** | **2.32e-2** | **2.80e-2** | **1.29e-4** | **1.77e-2** | **1.08e-2** |
|  | T6 | **n.t.** | **2.83e-3** | **8.56e-3** | **n.t.** | **1.97e-3** | **2.18e-3** |
|  | T12 | **n.t.** | **2.17e-3** | **4.49e-3** | **n.t.** | **2.75e-4** | **1.71e-2** |
| **DPS5** | T1 | **0.77e-4** | **1.39e-3** | 6.14e-2 | **3.04e-3** | **8.21e-3** | 0.18 |
|  | T3 | **2.34e-4** | **1.29e-2** | **2.87e-2** | **1.66e-4** | **3.36e-2** | **2.31e-3** |
|  | T6 | **n.t.** | **1.52e-3** | **4.11e-2** | **n.t.** | **1.44e-3** | **2.90e-2** |
|  | T12 ^exp^ | **n.t.** | **n.t.** | **2.03e-2** | **n.t.** | **0.47e-4** | **7.77e-4** |
| **DPS6** | T1 | **2.74e-3** | 0.14 | 0.23 | **6.14e-3** | **1.26e-2** | **7.80e-3** |
|  | T3 | **1.10e-2** | 0.27 | **2.33e-2** | **1.03e-2** | **4.09e-3** | **2.56e-3** |
|  | T6 | **3.60e-2** | **1.01e-2** | **1.45e-2** | **3.19e-4** | **5.98e-4** | **5.62e-3** |
|  | T12 ^exp^ | **0.14e-4** | **3.88e-4** | **1.48e-3** | **0.05e-4** | **3.62e-3** | **4.26e-3** |
| **DPS7** | T1 | **1.43e-3** | 0.38 | 5.61e-2 | **4.64e-4** | **1.15e-3** | **1.87e-3** |
|  | T3 | **3.79e-4** | 0.10 | **1.15e-3** | **4.59e-4** | **5.96e-4** | **8.58e-3** |
|  | T6 | **0.21e-4** | 0.13 | **1.39e-4** | **n.t.** | **n.t.** | **0.59e-4** |
|  | T12 | **n.t.** | **1.03e-3** | **1.41e-3** | **n.t.** | **n.t.** | **n.t.** |

**Supplementary Table C.3:** P-values and Z‑values in Friedman test followed by pairwise Wilcoxon signed-rank post-hoc tests. Statistical significance of pairwise comparisons was assessed using a Bonferroni-adjusted significance level (p=0.0083). (Comparison of bifidobacterial counts across storage temperatures at different time points.)

A) **Ambient**

Time points are defined as follows: T1, 1 month; T3, 3 months; T6, 6 months; and T12, 12 months of storage.

|  | T1 | T3 | T6 | T12 |
| --- | --- | --- | --- | --- |
| T1 |  | Z=2.37, p=0.02 | Z=2.37, p=0.02 | Z=2.37, p=0.02 |
| T3 | Z=2.37, p=0.02 |  | Z=2.37, p=0.02 | Z=2.37, p=0.02 |
| T6 | Z=2.37, p=0.02 | Z=2.37, p=0.02 |  | Z=1.60 p=0.11 |
| T12 | Z=2.37, p=0.02 | Z=2.37, p=0.02 | Z=1.60 p=0.11 |  |

B) **Refrigeration**

Time points are defined as follows: T1, 1 month; T3, 3 months; T6, 6 months; and T12, 12 months of storage.

|  | **T1** | **T3** | **T6** | **T12** |
| --- | --- | --- | --- | --- |
| **T1** |  | Z=2.37, p=0.02 | Z=2.37, p=0.02 | Z=2.37, p=0.02 |
| **T3** | Z=2.37, p=0.02 |  | Z=2.37, p=0.02 | Z=2.37, p=0.02 |
| **T6** | Z=2.37, p=0.02 | Z=2.37, p=0.02 |  | Z=2.37, p=0.02 |
| **T12** | Z=2.37, p=0.02 | Z=2.37, p=0.02 | Z=2.37, p=0.02 |  |

C) **Freezing**

Time points are defined as follows: T1, 1 month; T3, 3 months; T6, 6 months; and T12, 12 months of storage.

|  | **T1** | **T3** | **T6** | **T12** |
| --- | --- | --- | --- | --- |
| **T1** |  | Z=2.37, p=0.02 | Z=2.37, p=0.02 | Z=2.37, p=0.02 |
| **T3** | Z=2.37, p=0.02 |  | Z=2.37, p=0.02 | Z=2.37, p=0.02 |
| **T6** | Z=2.37, p=0.02 | Z=2.37, p=0.02 |  | Z=2.37, p=0.02 |
| **T12** | Z=2.37, p=0.02 | Z=2.37, p=0.02 | Z=2.37, p=0.02 |  |

**Supplementary Table C.4:** P-values and Z‑values in Kruskal–Wallis test followed by Dunn’s post‑hoc test with Bonferroni correction. (Comparison of bifidobacterial counts across all time points, independent of storage temperature.)

Time points are defined as follows: T1, 1 month; T3, 3 months; T6, 6 months; and T12, 12 months of storage.

|  | **Ambient_T1** | **Ambient_T3** | **Ambient_T6** | **Ambient_T12** | **Refrigeration_T1** | **Refrigeration_T3** | **Refrigeration_T6** | **Refrigeration_T12** | **Freezing_T1** | **Freezing_T3** | **Freezing_T6** | **Freezing_T12** |
| --- | --- | --- | --- | --- | --- | --- | --- | --- | --- | --- | --- | --- |
| **Ambient_**  **T1** |  | Z=1.50, p=1.00 | Z=2.12, p=1.00 | Z=2.50, p=0.81 | Z=1,17, p=1.00 | Z=0.78, p=1.00 | Z=1.64e-2, p=1.00 | Z=0.86, p=1.00 | Z=1.07, p=1.00 | Z=0.80, p=1.00 | Z=0.14, p=1.00 | Z=0.82, p=1.00 |
| **Ambient_**  **T3** | Z=1.50, p=1.00 |  | Z=0.62, p=1.00 | Z=1.01, p=1.00 | Z=2.66, p=0.51 | Z=2.28, p=1.00 | Z=1.51, p=1.00 | Z=0.64, p=1.00 | Z=2.57, p=0.67 | Z=2.30, p=1.00 | Z=1.64, p=1.00 | Z=0.68, p=1.00 |
| **Ambient_**  **T6** | Z=2.12, p=1.00 | Z=0.62, p=1.00 |  | Z=0.38, p=1.00 | Z=3.29, p=6.68e-2 | Z=2.90, p=0.24 | Z=2.14, p=1.00 | Z=1.26, p=1.00 | Z=3.19, p=9.27e-2 | Z=2.92, p=0.23 | Z=2.26, p=1.00 | Z=1.30, p=1.00 |
| **Ambient_**  **T12** | Z=2.50, p=0.81 | Z=1.01, p=1.00 | Z=0.38, p=1.00 |  | Z=3.67, p=1.60e-2 | Z=3.29, p=6.68e-2 | Z=2.52, p=0.77 | Z=1.64, p=1.00 | Z=3.58, p=2.29e-2 | Z=3.30, p=6.30e-2 | Z=2.65, p=0.54 | Z=1.69, p=1.00 |
| **Refrigeration_T1** | Z=1.17, p=1.00 | Z=2.66, p=0.51 | Z=3.29, p=6.68e-2 | Z=3.67, p=1.60e-2 |  | Z=0.38, p=1.00 | Z=1.15, p=1.00 | Z=2.03, p=1.00 | Z=9.31e-2, p=1.00 | Z=0.37, p=1.00 | Z=1.02, p=1.00 | Z=1.98, p=1.00 |
| **Refrigeration_T3** | Z=0.78, p=1.00 | Z=2.28, p=1.00 | Z=2.90, p=0.24 | Z=3.29, p=6.68e-2 | Z=0.38, p=1.00 |  | Z=0.77, p=1.00 | Z=1.64, p=1.00 | Z=0.29, p=1.00 | Z=1.64e-2, p=1.00 | Z=0.64, p=1.00 | Z=1.60, p=1.00 |
| **Refrigeration_T6** | Z=1.64e-2, p=1.00 | Z=1.51, p=1.00 | Z=2.14, p=1.00 | Z=2.52, p=0.77 | Z=1.15, p=1.00 | Z=0.77, p=1.00 |  | Z=0.88, p=1.00 | Z=1.06, p=1.00 | Z=0.78, p=1.00 | Z=0.13, p=1.00 | Z=0.83, p=1.00 |
| **Refrigeration_T12** | Z=0.86, p=1.00 | Z=0.64, p=1.00 | Z=1.26, p=1.00 | Z=1.64, p=1.00 | Z=2.03, p=1.00 | Z=1.64, p=1.00 | Z=0.88, p=1.00 |  | Z=1.93, p=1.00 | Z=1.66, p=1.00 | Z=1.00, p=1.00 | Z=4.38e-2, p=1.00 |
| **Freezing_**  **T1** | Z=1.07, p=1.00 | Z=2.57, p=0.67 | Z=3.19, p=9.27e-2 | Z=3.58, p=2.29e-2 | Z=9.31e-2, p=1.00 | Z=0.29, p=1.00 | Z=1.06, p=1.00 | Z=1.93, p=1.00 |  | Z=0.27, p=1.00 | Z=0.93, p=1.00 | Z=1.89, p=1.00 |
| **Freezing_**  **T3** | Z=0.80, p=1.00 | Z=2.30, p=1.00 | Z=2.92, p=0.23 | Z=3.30, p=6.30e-2 | Z=0.37, p=1.00 | Z=1.64e-2, p=1.00 | Z=0.78, p=1.00 | Z=1.66, p=1.00 | Z=0.27, p=1.00 |  | Z=0.66, p=1.00 | Z=1.62, p=1.00 |
| **Freezing_**  **T6** | Z=0.14, p=1.00 | Z=1.64, p=1.00 | Z=2.26, p=1.00 | Z=2.65, p=0.54 | Z=1.02, p=1.00 | Z=0.64, p=1.00 | Z=0.13, p=1.00 | Z=1.00, p=1.00 | Z=0.93, p=1.00 | Z=0.66, p=1.00 |  | Z=0.96, p=1.00 |
| **Freezing_**  **T12** | Z=0.82, p=1.00 | Z=0.68, p=1.00 | Z=1.30, p=1.00 | Z=1.69, p=1.00 | Z=1.98, p=1.00 | Z=1.60, p=1.00 | Z=0.83, p=1.00 | Z=4.38e-2, p=1.00 | Z=1.89, p=1.00 | Z=1.62, p=1.00 | Z=0.96, p=1.00 |  |

**Supplementary Table C.5:** P-values and Z‑values in Friedman test followed by pairwise Wilcoxon signed-rank post-hoc tests. Statistical significance of pairwise comparisons was assessed using a Bonferroni-adjusted significance level (p=0.0083). (Comparison of lactobacilli counts across storage temperatures at different time points.)

A) **Ambient**

Time points are defined as follows: T1, 1 month; T3, 3 months; T6, 6 months; and T12, 12 months of storage.

|  | **T1** | **T3** | **T6** | **T12** |
| --- | --- | --- | --- | --- |
| **T1** |  | Z=2.20, p=0.03 | Z=2.37, p=0.02 | Z=2.37, p=0.02 |
| **T3** | Z=2.20, p=0.03 |  | Z=2.37, p=0.02 | Z=2.37, p=0.02 |
| **T6** | Z=2.37, p=0.02 | Z=2.37, p=0.02 |  | Z=1.34, p=0.18 |
| **T12** | Z=2.37, p=0.02 | Z=2.37, p=0.02 | Z=1.34, p=0.18 |  |

B) **Refrigeration**

Time points are defined as follows: T1, 1 month; T3, 3 months; T6, 6 months; and T12, 12 months of storage.

|  | **T1** | **T3** | **T6** | **T12** |
| --- | --- | --- | --- | --- |
| **T1** |  | Z=2.37, p=0.02 | Z=2.37, p=0.02 | Z=2.37, p=0.02 |
| **T3** | Z=2.37, p=0.02 |  | Z=2.37, p=0.02 | Z=2.37, p=0.02 |
| **T6** | Z=2.37, p=0.02 | Z=2.37, p=0.02 |  | Z=2.20, p=0.03 |
| **T12** | Z=2.37, p=0.02 | Z=2.37, p=0.02 | Z=2.20, p=0.03 |  |

C) **Freezing**

Time points are defined as follows: T1, 1 month; T3, 3 months; T6, 6 months; and T12, 12 months of storage.

|  | **T1** | **T3** | **T6** | **T12** |
| --- | --- | --- | --- | --- |
| **T1** |  | Z=2.37, p=0.02 | Z=2.37, p=0.02 | Z=2.37, p=0.02 |
| **T3** | Z=2.37, p=0.02 |  | Z=2.37, p=0.02 | Z=2.37, p=0.02 |
| **T6** | Z=2.37, p=0.02 | Z=2.37, p=0.02 |  | Z=2.37, p=0.02 |
| **T12** | Z=2.37, p=0.02 | Z=2.37, p=0.02 | Z=2.37, p=0.02 |  |

**Supplementary Table C.6:** P-values and Z‑values in Kruskal–Wallis test followed by Dunn’s post‑hoc test with Bonferroni correction. (Comparison of lactobacilli counts across all time points, independent of storage temperature.)

Time points are defined as follows: T1, 1 month; T3, 3 months; T6, 6 months; and T12, 12 months of storage.

|  | **Ambient_T1** | | **Ambient_T3** | | **Ambient_T6** | | **Ambient_T12** | | **Refrigerati**  **on_T1** | | **Refrigerati**  **on_T3** | | **Refrigerati**  **on_T6** | | **Refrigerati**  **on_T12** | | **Freezing_T1** | | **Freezing_T3** | | **Freezing_T6** | | **Freezing_T12** | |
| --- | --- | --- | --- | --- | --- | --- | --- | --- | --- | --- | --- | --- | --- | --- | --- | --- | --- | --- | --- | --- | --- | --- | --- | --- |
| **Ambient_**  **T1** |  | Z=1.56, p=1.00 | | Z=2.47, p=0.89 | | Z=2.75, p=0.39 | | Z=0.58, p=1.00 | | Z=0.12, p=1.00 | | Z=0.64e-2, p=1.00 | | Z=1.30, p=1.00 | | Z=0.50, p=1.00 | | Z=0.25, p=1.00 | | Z=2.19e-2, p=1.00 | | Z=0.66, p=1.00 | |  |
| **Ambient_**  **T3** | Z=1.56, p=1.00 |  | | Z=0.91, p=1.00 | | Z=1.19, p=1.00 | | Z=2.14, p=0.51 | | Z=1.68, p=1.00 | | Z=0.93, p=1.00 | | Z=0.26, p=1.00 | | Z=2.07, p=1.00 | | Z=1.81, p=1.00 | | Z=1.54, p=1.00 | | Z=0.90, p=1.00 | |  |
| **Ambient_**  **T6** | Z=2.47, p=0.89 | Z=0.91, p=1.00 | |  | | Z=0.28, p=1.00 | | Z=3.05, p=0.15 | | Z=2.59, p=0.64 | | Z=1.84, p=1.00 | | Z=1.17, p=1.00 | | Z=2.97, p=0.19 | | Z=2.72, p=0.43 | | Z=2.45, p=0.95 | | Z=1.81, p=1.00 | |  |
| **Ambient_**  **T12** | Z=2.75, p=0.39 | Z=1.19, p=1.00 | | Z=0.28, p=1.00 | |  | | Z=3.33, p=5.71e-2 | | Z=2.87, p=0.28 | | Z=2.11, p=1.00 | | Z=1.45, p=1.00 | | Z=3.25, p=7.51e-2 | | Z=3.00, p=0.18 | | Z=2.73, p=0.42 | | Z=2.09, p=1.00 | |  |
| **Refrigeration_T1** | Z=0.58, p=1.00 | Z=2.14, p=0.51 | | Z=3.05, p=0.15 | | Z=3.33, p=5.71e-2 | |  | | Z=0.47, p=1.00 | | Z=1.22, p=1.00 | | Z=1.88, p=1.00 | | Z=7.67e-2, p=1.00 | | Z=0.33, p=1.00 | | Z=0.60, p=1.00 | | Z=1.24, p=1.00 | |  |
| **Refrigeration_T3** | Z=0.12, p=1.00 | Z=1.68, p=1.00 | | Z=2.59, p=0.64 | | Z=2.87, p=0.28 | | Z=0.47, p=1.00 | |  | | Z=0.75, p=1.00 | | Z=1.42, p=1.00 | | Z=0.39, p=1.00 | | Z=0.14, p=1.00 | | Z=0.14, p=1.00 | | Z=0.78, p=1.00 | |  |
| **Refrigeration_T6** | Z=0.64e-2, p=1.00 | Z=0.93, p=1.00 | | Z=1.84, p=1.00 | | Z=2.11, p=1.00 | | Z=1.22, p=1.00 | | Z=0.75, p=1.00 | |  | | Z=0.67, p=1.00 | | Z=1.14, p=1.00 | | Z=0.89, p=1.00 | | Z=0.61, p=1.00 | | Z=2.74e-2, p=1.00 | |  |
| **Refrigeration_T12** | Z=1.30, p=1.00 | Z=0.26, p=1.00 | | Z=1.17, p=1.00 | | Z=1.45, p=1.00 | | Z=1.88, p=1.00 | | Z=1.42, p=1.00 | | Z=0.67, p=1.00 | |  | | Z=1.81, p=1.00 | | Z=1.56, p=1.00 | | Z=1.28, p=1.00 | | Z=0.64, p=1.00 | |  |
| **Freezing_**  **T1** | Z=0.50, p=1.00 | Z=2.07, p=1.00 | | Z=2.97, p=0.19 | | Z=3.25, p=7.51e-2 | | Z=7.67e-2, p=1.00 | | Z=0.39, p=1.00 | | Z=1.14, p=1.00 | | Z=1.81, p=1.00 | |  | | Z=0.25, p=1.00 | | Z=0.53, p=1.00 | | Z=1.17, p=1.00 | |  |
| **Freezing_**  **T3** | Z=0.25, p=1.00 | Z=1.81, p=1.00 | | Z=2.72, p=0.43 | | Z=3.00, p=0.18 | | Z=0.33, p=1.00 | | Z=0.14, p=1.00 | | Z=0.89, p=1.00 | | Z=1.56, p=1.00 | | Z=0.25, p=1.00 | |  | | Z=0.27, p=1.00 | | Z=0.91, p=1.00 | |  |
| **Freezing_**  **T6** | Z=2.19e-2, p=1.00 | Z=1.54, p=1.00 | | Z=2.45, p=0.95 | | Z=2.73, p=0.42 | | Z=0.60, p=1.00 | | Z=0.14, p=1.00 | | Z=0.61, p=1.00 | | Z=1.28, p=1.00 | | Z=0.53, p=1.00 | | Z=0.27, p=1.00 | |  | | Z=0.64, p=1.00 | |  |
| **Freezing_**  **T12** | Z=0.66, p=1.00 | Z=0.90, p=1.00 | | Z=1.81, p=1.00 | | Z=2.09, p=1.00 | | Z=1.24, p=1.00 | | Z=0.78, p=1.00 | | Z=2.74e-2, p=1.00 | | Z=0.64, p=1.00 | | Z=1.17, p=1.00 | | Z=0.91, p=1.00 | | Z=0.64, p=1.00 | |  | |  |
